# LKB1 tumor suppressor and salt-inducible kinases negatively regulate human T-cell leukemia virus type 1 transcription

**DOI:** 10.1186/1742-4690-10-40

**Published:** 2013-04-11

**Authors:** Hei-Man Vincent Tang, Wei-Wei Gao, Ching-Ping Chan, Yeung-Tung Siu, Chi-Ming Wong, Kin-Hang Kok, Yick-Pang Ching, Hiroshi Takemori, Dong-Yan Jin

**Affiliations:** 1 Department of Biochemistry, The University of Hong Kong, 21 Sassoon Road, Pokfulam, Hong Kong; 2 Department of Anatomy, The University of Hong Kong, 21 Sassoon Road, Pokfulam, Hong Kong; 3 Laboratory of Cell Signaling and Metabolism, National Institute of Biomedical Innovation, Osaka, Japan

**Keywords:** Adult T-cell leukemia, Human T-cell leukemia virus type 1, LKB1 tumor suppressor, Salt-inducible kinases, Metformin, Tax oncoprotein

## Abstract

**Background:**

Human T-cell leukemia virus type 1 (HTLV-1) causes adult T-cell leukemia (ATL). Treatment options are limited and prophylactic agents are not available. We have previously demonstrated an essential role for CREB-regulating transcriptional coactivators (CRTCs) in HTLV-1 transcription.

**Results:**

In this study we report on the negative regulatory role of LKB1 tumor suppressor and salt-inducible kinases (SIKs) in the activation of HTLV-1 long terminal repeats (LTR) by the oncoprotein Tax. Activation of LKB1 and SIKs effectively blunted Tax activity in a phosphorylation-dependent manner, whereas compromising these kinases, but not AMP-dependent protein kinases, augmented Tax function. Activated LKB1 and SIKs associated with Tax and suppressed Tax-induced LTR activation by counteracting CRTCs and CREB. Enforced expression of LKB1 or SIK1 in cells transfected with HTLV-1 molecular clone pX1MT repressed proviral transcription. On the contrary, depletion of LKB1 in pX1MT-transfected cells and in HTLV-1-transformed T cells boosted the expression of Tax. Treatment of HTLV-1 transformed cells with metformin led to LKB1/SIK1 activation, reduction in Tax expression, and inhibition of cell proliferation.

**Conclusions:**

Our findings revealed a new function of LKB1 and SIKs as negative regulators of HTLV-1 transcription. Pharmaceutical activation of LKB1 and SIKs might be considered as a new strategy in anti-HTLV-1 and anti-ATL therapy.

## Background

HTLV-1 was the first human retrovirus shown to be the etiological agent of adult T-cell leukemia (ATL). Over 20 million people are infected with HTLV-1 worldwide. ATL is a highly aggressive and fatal malignancy of CD4^+^ T lymphocytes that develops in 2-5% of carriers, usually after more than 20 years of HTLV-1 latency [1,2]. Although it is a slow and multifactorial process, progression of ATL tightly correlates with high HTLV-1 proviral load [3]. Treatments for ATL are unspecific and unsatisfactory. Once developed, ATL is minimally treatable. In addition, prophylactic agents that can prevent the development of ATL in carriers of HTLV-1 remain to be identified [2].

Expression of HTLV-1 provirus is transcriptionally mediated through the viral transactivator Tax, which potently stimulates the activity of long terminal repeats (LTR) by activating the cellular transcription factor CREB and coactivators such as CREB-binding protein (CBP) and CREB-regulating transcriptional coactivators (CRTCs), also known as transducers of regulated CREB activity (TORCs) [4-9]. We have previously characterized the essential roles of Tax, CREB and CRTCs in this process [7,10,11]. Particularly, we have demonstrated the requirement of CRTCs in Tax activation of the LTR [7].

In addition to a viral LTR, a battery of cellular genes can also be activated by Tax primarily through CREB and NF-κB. Tax is a major viral oncoprotein that plays a crucial role in the initiation of malignant transformation [12]. Tax also cooperates with other viral oncoproteins such as HBZ in later phases of leukemogenesis [13]. It has been generally accepted that activation of both CREB and NF-κB by Tax is required for full-blown transformation [12].

Consistent with Tax playing an essential role in both viral transcription and oncogenesis, counteracting Tax function or eliminating Tax-expressing cells has shown anti-HTLV-1 and anti-ATL effects [14,15]. Thus, we hypothesized that identification of protein kinases that regulate Tax might reveal new strategies for disease prevention and intervention using small-molecule agonists and antagonists of such kinases. In searching for these kinases, we noticed that CRTCs are regulated by upstream kinases such as LKB1, AMP-activated protein kinases (AMPKs) and salt-inducible kinases (SIKs) [16-18]. LKB1 is a tumor suppressor inactivated in Peutz-Jeghers syndrome, a rare autosomal dominant disorder characterized by gastrointestinal polyps and a higher risk of malignancy [19]. LKB1 phosphorylates and activates AMPKs and AMPK-related kinases, which in turn phosphorylate CRTC2 at S171, leading to its association with 14-3-3 proteins and sequestration in the cytoplasm [16]. As such, AMPKs and SIKs are major kinases that regulate CRTCs [18,20]. However, the roles of LKB1, AMPKs and SIKs in Tax-mediated transcriptional activation remain elusive.

In this study, we investigated the regulatory roles of LKB1, AMPKs and SIKs in Tax activation of the HTLV-1 LTR. We demonstrated LKB1 and SIKs to be negative regulators of HTLV-1 transcription. Our work suggests a new model in which LKB1 and SIKs suppress Tax-mediated LTR activation by targeting CRTCs and CREB. Our findings also implicate the utility of small-molecule agonists of LKB1 and SIKs in anti-HTLV-1 and anti-ATL therapy.

## Results

### LKB1 inhibits Tax activation of LTR in a kinase-dependent manner

Four lines of analysis prompted us to investigate the role of LKB1 in HTLV-1 transcription. First, chromosomal rearrangements at 19p13.3, where LKB1 is located, have been reported in some ATL cells [21]. Second, it will be of interest to see whether LKB1 is another repressor of HTLV-1 replication like p53 and p30^II^, which play a role in viral persistence and transformation [22,23]. Third, small-molecule agonists of LKB1 have been extensively tested as targeted therapeutics [19]. Finally, Tax-dependent transcriptional activation is particularly robust in LKB1-null HeLa cells.

**Figure 1:**
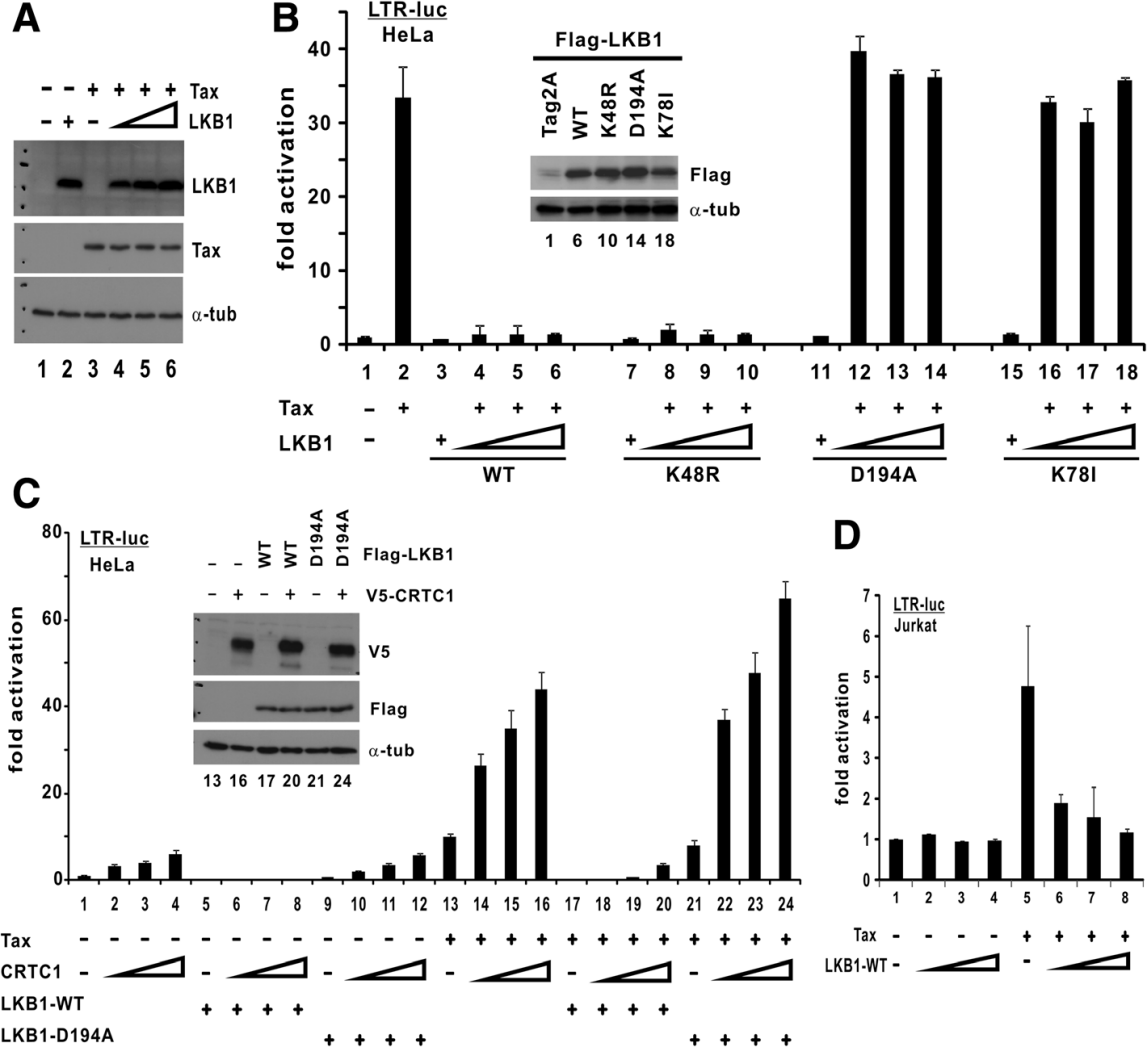
**LKB1 suppresses Tax-mediated LTR activation in a kinase-dependent manner.** (**A**) Expression of LKB1 and Tax. HeLa cells were transfected with pIEX, pCMV-Tag2-LKB1, or pIEX plus escalating amounts of pCMV-Tag2-LKB1. Cell lysates were probed with mouse anti-Tax, mouse anti-Flag and mouse anti-α-tubulin (α-tub). (**B**) Kinase activity of LKB1 is required for suppression of Tax activity. HeLa cells were transfected with pLTR-Luc (100 ng), a fixed amount of pIEX (100 ng) and escalating amounts of LKB1 plasmids (25, 50 and 100 ng). Protein expression was analyzed (inset). Fold activation is calculated from pLTR-Luc activity normalized to that of pSV-RLuc. Results of this dual luciferase assay represent means from three independent experiments and error bars indicate SD. The differences between groups 2 and 6, and between groups 2 and 10 are statistically significant by two tailed Student’s t test (p = 0.00020 for both). In contrast, there is no statistically significant difference between groups 2 and 14 (p = 0.34) or between groups 2 and 18 (p = 0.29). (**C**) Kinase-dependent suppression of CRTC1 and Tax by LKB1. HeLa cells were transfected with pLTR-Luc (100 ng), a fixed amount of pIEX (100 ng) plus pCMV-Tag2-LKB1 (WT or D194A) (30 ng), and escalating amounts of expression plasmids (18, 37 and 75 ng) for CRTC1. Statistically significant differences are found between groups 4 and 8 (p = 0.00097) and between groups 16 and 20 (p = 0.000035). (**D**) LKB1 inhibition of LTR in T cells. Jurkat cells were transfected with pLTR-Luc (300 ng), a fixed amount of pCAG-Tax-V5 (200 ng) and escalating amounts of LKB1 plasmid (50, 100 and 200 ng). The difference between group 5 and groups 6–8 is statistically significant (p = 0.00084).

To determine whether LKB1 might inhibit Tax activity, we coexpressed LKB1 and Tax in HeLa cells (Figure 1A). The expression of Tax was driven by a CMV promoter and the level of Tax protein remained unchanged when the dose of LKB1 was escalated (Figure 1A, lane 3 compared to 4–6). In the presence of Tax, LKB1 effectively abolished LTR-driven luciferase reporter expression even at the lowest dose (Figure 1B, lane 2 compared to 4–6). Similar results were also obtained with LKB1-K48R (Figure 1B, lanes 7–10), a dominant active mutant [24]. On the contrary, two kinase-dead mutants [25,26], LKB1-D194A and LKB1-K78I, had no influence on Tax activation of LTR (Figure 1B, lanes 11–14 and 15–18).

LKB1 negatively regulates CREB-mediated transcription through AMPK- and SIK-dependent inhibitory phosphorylation of CRTCs [27,28], which are also essential coactivators of Tax [7]. We therefore further asked whether LKB1 might exert any effects on CRTC1 coactivation of Tax. When we coexpressed LKB1 and CRTC1 in HeLa cells (Figure 1C inset), we found that wild-type LKB1 (LKB1-WT), but not its kinase-defective mutant LKB1-D194A, abrogated LTR activation either by CRTC1 alone (Figure 1C, lanes 1–4 compared to 5–8 or 9–12) or by CRTC1 and Tax (Figure 1C, lanes 13–16 compared to 17–20 or 21–24). To verify the suppressive effect of LKB1 on Tax in T lymphocytes, which are the target cells of HTLV-1, we expressed LKB1 in Jurkat cells. We observed less pronounced activation of LTR by Tax in LKB1-proficient Jurkat cells compared to LKB1-null HeLa cells (Figure 1B); however, introduction of exogenous LKB1 resulted in further inhibition of Tax activation of LTR (Figure 1D, lanes 1–4 compared to 5–8). Hence, LKB1 suppresses Tax- and CRTC-mediated activation of HTLV-1 LTR in a kinase-dependent manner.

### SIKs inhibit Tax activation of LTR in a kinase-dependent manner

The role of AMPKs in phosphorylation and activation of CRTCs has been documented in worms and humans [28,29]. In addition, SIKs [30], which were identified in the adrenal glands of rats fed with high salt diet and are also phosphorylated by LKB1, are alternative upstream regulators of CRTCs in CREB signaling [31]. With this in mind, we sought to determine the role of AMPKs and SIKs in Tax activation of LTR.

**Figure 2:**
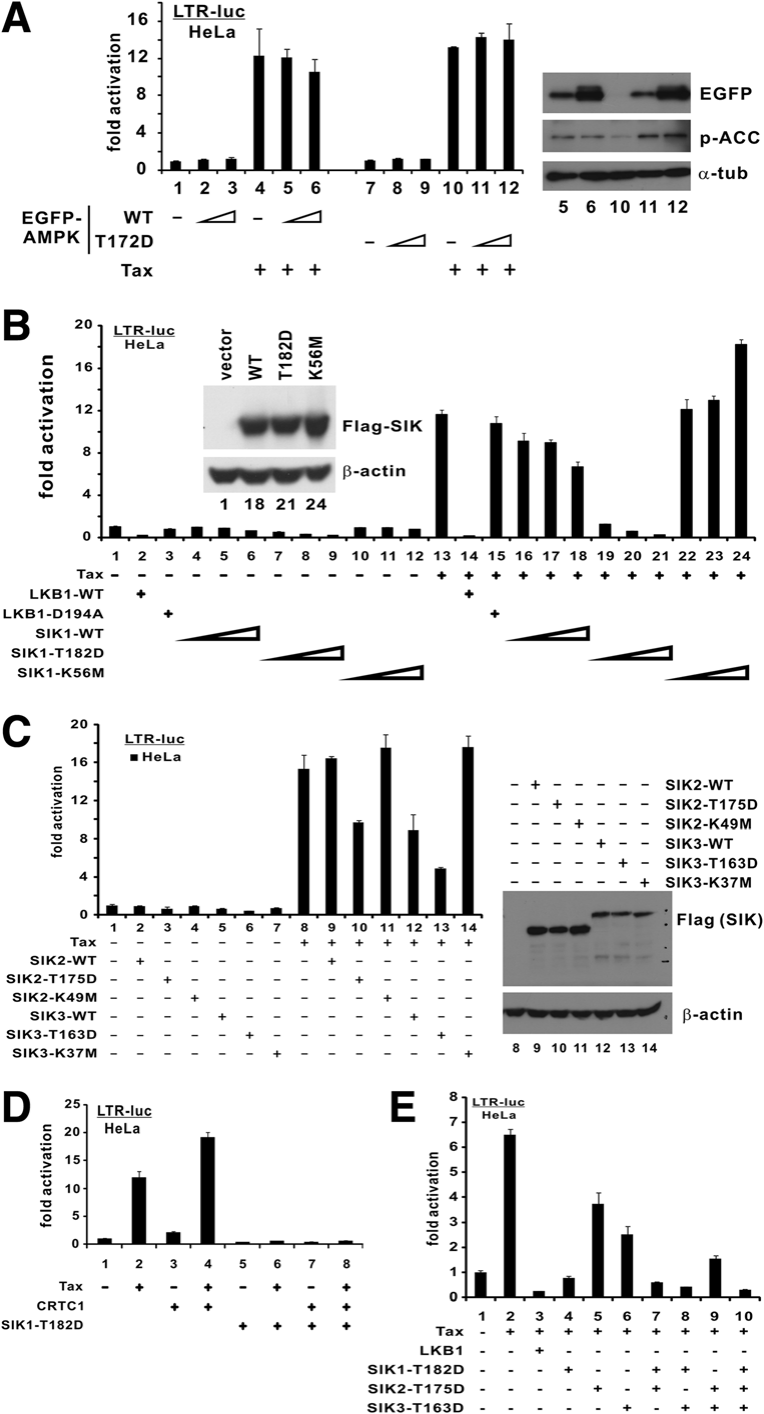
**SIKs suppress Tax-mediated LTR activation in a kinase-dependent manner.** (**A**) Influence of AMPK. HeLa cells were transfected with pLTR-Luc (100 ng), a fixed amount of pIEX (100 ng) and escalating amounts of AMPK plasmids (25, 50 and 100 ng). Endogenous p-ACC indicates AMPK activity. Two tailed Student's t test was performed (groups 4 and 6: p = 0.16; groups 10 and 12: p = 0.30). (**B**) Kinase activity of SIK1 is required for suppression. HeLa cells were transfected with pLTR-Luc (100 ng), a fixed amount of pIEX (100 ng) plus pCMV-Tag2-LKB1-WT/D194A (30 ng), and escalating amounts of SIK1 plasmids (25, 50 and 100 ng). p values were calculated for selected groups (13 and 18: 0.00038; 13 and 21: 0.0000030; 13 and 24: 0.000050). (**C**) Kinase activity of SIK2/3 is required for suppression. HeLa cells were transfected with pLTR-Luc (100 ng), a fixed amount of pIEX (100 ng) and pCMV-Tag2-SIK2-WT/T175D/K49M (30 ng) or pCMV-Tag2-SIK3-WT/T163D/K37M (30 ng). p values were calculated for selected groups (8 and 10: 0.0050: 8 and 12: 0.010; 8 and 13: 0.00030; 8 and 11: 0.25; 8 and 14: 0.37). (**D**) Suppression of CRTC1- and Tax-mediated LTR activation by activated SIK1. HeLa cells were transfected with pLTR-Luc (100 ng) and a fixed amount of pIEX (100 ng) and pcDNA3.1/V5-CRTC1 (18 ng) or pCMV-Tag2-SIK1-T182D (50 ng). The difference between groups 4 and 8 is statistically significant (p = 0.000054). (**E**) Cooperation of activated SIK1/2/3. A fixed amount of pIEX (100 ng) and a fixed total amount (30 ng) of pCMV-Tag2-LKB1-WT, pCMV-Tag2-SIK1-T182D, pCMV-Tag2-SIK2-T175D, pCMV-Tag2-SIK3-T163D or their indicated combinations were transfected into HeLa cells. p values were calculated for selected groups (4 and 7: 0.0066; 4 and 8: 0.00046; 7 and 10: 0.00016; 8 and 10: 0.00017).

When we expressed EGFP-AMPKα2-WT and its constitutively active mutant (T172D) in HeLa cells, Tax-mediated activation of LTR was unaffected (Figure 2A, lanes 4 compared to 5 and 6; and lane 10 compared to 11 and 12). The expression and activity of EGFP-AMPKα2 proteins in HeLa cells were verified by immunoblotting. Detection of phosphorylated ACC, a known substrate of AMPK, indicated that the EGFP-AMPKα2 proteins expressed are catalytically active (Figure 2A, right panel, lanes 10 compared to 5, 6, 11 and 12). Because AMPKα2 is highly representative of the AMPK family [19], these results suggested that AMPK is unlikely involved in Tax activation of LTR.

We next examined the influence of SIK1 on Tax activity. SIK1-WT and its constitutively active (T182D) and kinase-defective (K56M) mutants were found to be expressed to comparable levels in HeLa cells (Figure 2B inset). The SIK1-T182D mutant was used to mimic the activation of SIK1 by LKB1 and was therefore expected to be more active than SIK1-WT [27,32]. Indeed, SIK1-T182D completely suppressed Tax activation of LTR (Figure 2B, lane 13 compared to 19–21), while SIK1-WT displayed only moderate suppressive effect (Figure 2B, lane 13 compared to 16–18). In contrast, SIK1-K56M even augmented Tax activity at the highest dose (Figure 2B, lane 13 compared to 22–24).

We further extended our analysis to SIK2 and SIK3. The respective constitutively active (SIK2-T175D and SIK3-T163D) and kinase-defective mutants (SIK2-K49M and SIK3-K37M) were expressed in HeLa cells (Figure 2C, right panel) [27,33]. It is noteworthy that SIK3-WT as well as constitutively active phosphomimetic mutants of SIK2 and SIK3 exhibited strong suppressive effects on Tax activity (Figure 2C, lane 8 compared to 10, 12 and 13). Since SIK2-WT did not inhibit Tax activity (Figure 2C, lane 9), the use of SIK1-T175D to mimic the activation by LKB1 was necessary. No alteration in Tax activation of LTR was observed for SIK2/3 kinase-defective mutants (Figure 2C, lane 8 compared to 11 and 14). These results were generally consistent with the notion that LKB1 phosphorylates and activates SIKs, which in turn phosphorylate and inhibit CRTCs.

To verify this model, we asked whether SIK1-T182D might counteract CRTC coactivation of Tax. Indeed, expression of SIK1-T182D ablated transcriptional activity of CRTC1 and Tax (Figure 2D, lanes 2–4 compared to 6–8), implicating SIKs as the potential targets of LKB1 in the activation of LTR by CRTCs and Tax. Furthermore, we coexpressed three active SIKs in HeLa cells. Interestingly, the inhibition of Tax activity was more robust when SIK1 + SIK2, SIK1 + SIK3 or SIK1 + SIK2 + SIK3 were expressed (Figure 2E, lanes 7, 8 and 10 compared to 4–6 and 9), suggesting that SIK1 might cooperate with SIK2 and SIK3 to suppress Tax activity subsequent to their activation by LKB1.

### Depletion of LKB1 or SIKs augments Tax activation of LTR

Above we have shown that the kinase-dead mutants of LKB1 and SIKs either have no influence on, or they enhance Tax activation of LTR in the LKB1-null HeLa cells. Our results implied that loss of LKB1 or SIK activity could de-repress the inhibition of Tax activity. Complementary to these gain-of-function analyses, here we further investigated the physiological importance of LKB1 and SIKs in Tax activation of LTR by compromising LKB1 and SIKs in HEK293T cells with RNAi.

**Figure 3:**
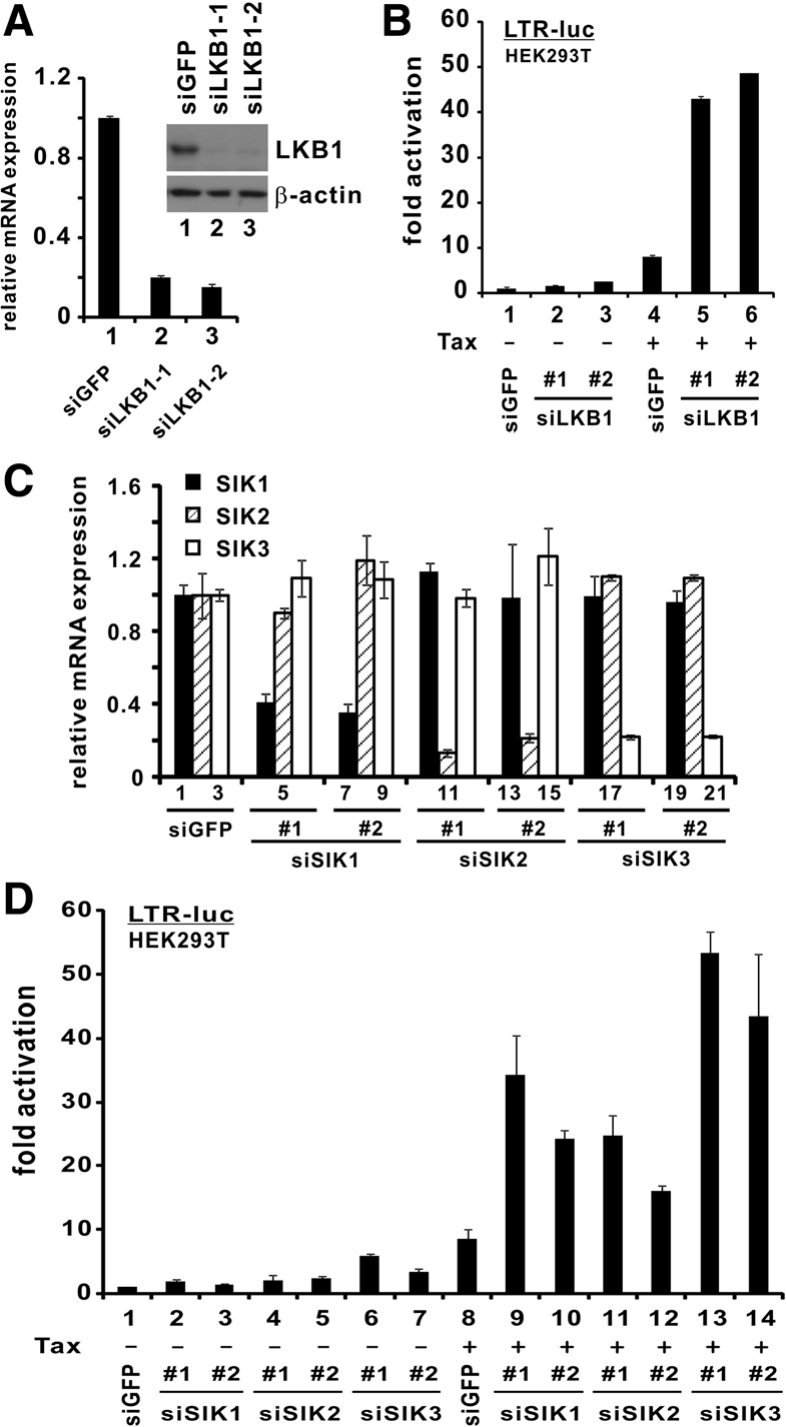
**Silencing of LKB1 and SIKs facilitates Tax activation of LTR.** (**A**) Verification of LKB1 knockdown. RT-qPCR was performed to analyze LKB1 and GAPDH transcripts. Quantitation of mRNA expression was achieved by comparative Ct method. Relative LKB1 mRNA expression in siGFP-transfected cells was taken as 1. Endogenous LKB1 in siRNA-transfected HEK293T cells was also analyzed by Western blotting at 72 hr post-transfection (inset). Two independent LKB1-targeting siRNAs (siLKB1-1 and siLKB1-2) at a concentration of 100 nM were used to deplete endogenous LKB1. siGFP was used as a negative control. Statistically significant differences exist between groups 1 and 2 or 1 and 3 (p = 0.0000033 or 0.0000073 by two tailed Student’s t test). (**B**) Downregulation of endogenous LKB1 augmented Tax activation of LTR. After 36 hrs of knockdown, plasmids pLTR-Luc, pSV-RLuc and pIEX were cotransfected into HEK293T cells. The difference between groups 4 and 5 or 4 and 6 is statistically significant (p = 0.0045 or 0.0080). (**C**) Verification of SIK knockdown by siRNAs. RT-qPCR was performed as in A. p values for selected bars were calculated (1 and 4: 0.00020; 1 and 7: 0.00013; 2 and 11: 0.00027; 2 and 14: 0.00042; 3 and 18: 0.0000046; 3 and 21: 0.00012). (**D**) Downregulation of individual endogenous SIK augmented Tax activity. Two independent siRNAs at a concentration of 100 nM were used to deplete endogenous SIKs in HEK293T cells. siGFP was used as a negative control. After 36 hrs of knockdown, plasmids pLTR-Luc, pSV-RLuc and pIEX were cotransfected into cells. Cells were harvested 36 hrs after the second transfection. Statistically significant differences exist between group 8 and each of groups 9–14 (p = 0.027, p = 0.001, p = 0.030, p = 0.0051, p = 0.0034 and p = 0.037, respectively).

LKB1 was abundantly expressed in HEK293T cells and the two independent siRNAs directed against LKB1 (siLKB1-1 and siLKB1-2) efficiently depleted its mRNA and protein expression (Figure 3A, lane 1 compared to 2 and 3). Correspondingly, Tax-mediated LTR activation was substantially potentiated in LKB1-depleted cells (Figure 3B, lane 4 compared to 5 and 6). Likewise, the effectiveness of two independent siRNAs targeting each of the SIKs in depleting their corresponding mRNA was validated (Figure 3C) and the potentiation of Tax activity was also observed in individual SIK-compromised cells (Figure 3D, lane 8 compared to 9–14). Thus, endogenous LKB1 and SIKs are physiological repressors of Tax function.

### Association of Tax with LKB1 and SIKs

For LKB1 and SIKs to exert their influence on Tax, they should form a protein complex with Tax inside the cell. To test this, we performed coimmunoprecipitation assays in HEK293T cells.

**Figure 4:**
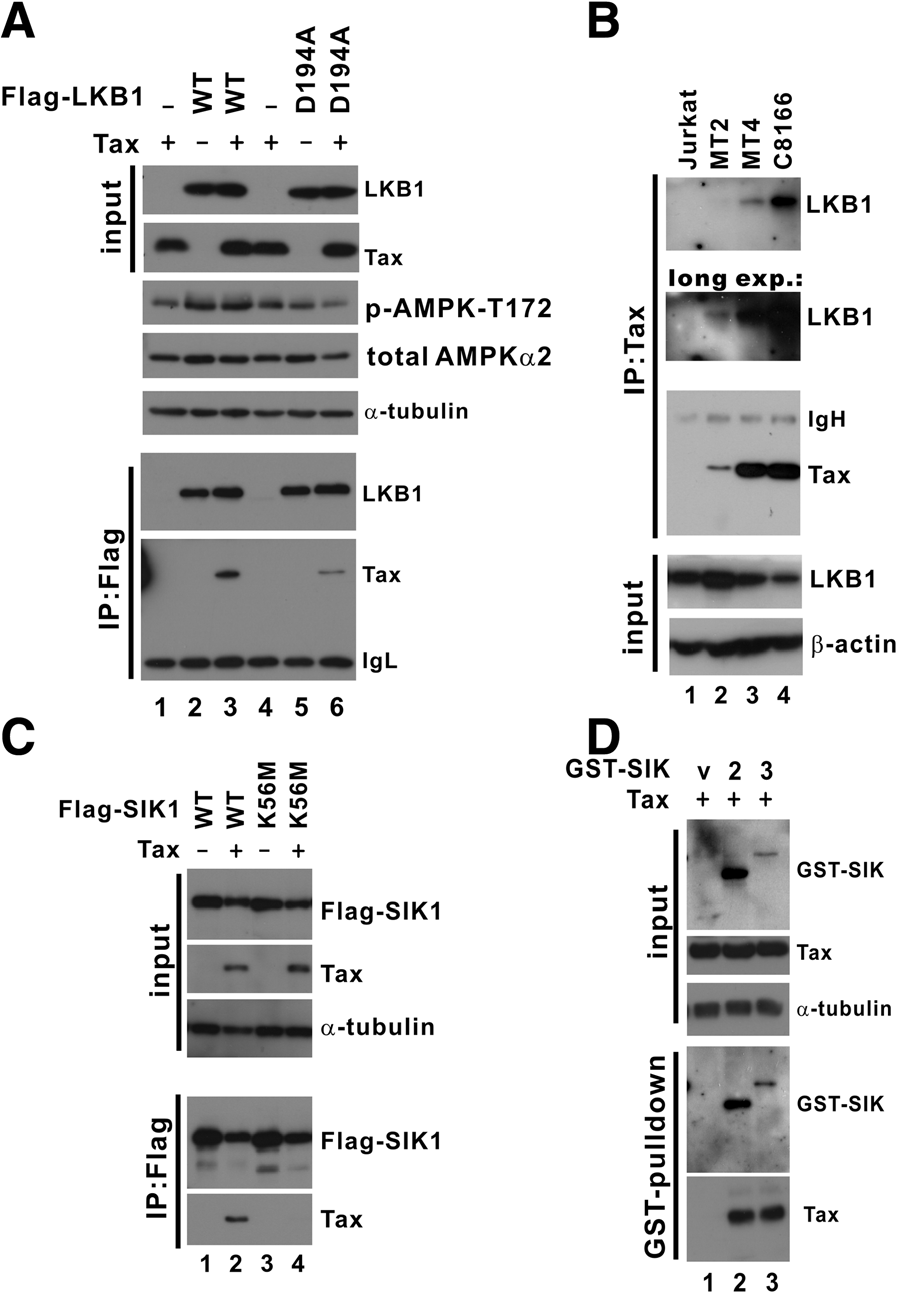
**Association of Tax with activated LKB1 and SIKs.** (**A**) Association with LKB1 in HEK293T cells. Cells were transfected with expression plasmids pCMV-Tag2-LKB1 (WT/D194A) and pCAG-Tax-V5. LKB1 was immunoprecipitated with anti-Flag. The precipitates were analyzed by Western blotting with anti-Flag and anti-Tax, respectively. The input lysates were also immunoblotted for LKB1, Tax and α-tubulin. Detection of phospho-AMPK-T172 (p-AMPK-T172) and total AMPKα2 indicated the kinase activity of LKB1. (**B**) Association with endogenous LKB1 in T cells. Jurkat, MT2, MT4 and C8166 cells were lysed and immunoprecipitated with anti-Tax. The precipitates were immunoblotted with anti-LKB1 and anti-Tax. A longer exposure (long exp.) of the LKB1 blot is also presented. The input lysates were analyzed for LKB1 and β-actin. (**C**) Association with SIK1. HEK293T cells were transfected with expression plasmids pCMV-Tag2-SIK1 (WT/K56M) and pCAG-Tax-V5. SIK1 was immunoprecipitated with anti-Flag. The precipitates were analyzed by Western blotting with anti-Flag and anti-Tax. The input lysates were also probed for SIK1, Tax and α-tubulin. (**D**) Association with SIK2 and SIK3. HEK293T cells were transfected with expression plasmids for pEBG vector (v), pEBG-SIK2 (2), pEBG-SIK3 (3) and pCAG-Tax-V5. GST-SIK2/3 was pulled down by glutathione-Sepharose 4B. The pull-down fraction was analyzed by Western blotting with anti-GST and anti-Tax. The input lysates were also probed for SIK2/3, Tax and α-tubulin.

A protein complex of Tax and LKB1 was detected in cells expressing both entities (Figure 4A, lane 3). This association between Tax and LKB1 was specific, as complex formation was not observed when either Tax or LKB1 alone was expressed (Figure 4A, lanes 1 and 2). Interestingly, the association between Tax and the kinase-dead LKB1-D194A mutant was considerably less pronounced than that between Tax and LKB1-WT (Figure 4A, lane 6 compared to 3). Thus, Tax might interact preferentially with active LKB1. The catalytic activity of LKB1-WT in cells was validated by probing AMPKs phosphorylated at T172. An elevation of phospho-AMPK was detected in cells expressing LKB1-WT, but not LKB1-D194A (Figure 4A, lanes 2 and 3 compared to 5 and 6). Notably, expression of Tax did not further enhance phosphorylation of AMPK by LKB1 (Figure 4A, lane 3 compared to lane 2). Consistent with this, an *in vitro* kinase assay with recombinant GST-AMPK, LKB1 and Tax proteins indicated that the addition of Tax did not significantly affect the kinase activity of LKB1 on AMPK (Additional file 1: Figure S1, lanes 3–5 compared to lane 2). In addition to HEK293T cells, HTLV-1-transformed T cells were also examined for the interaction between LKB1 and Tax. LKB1 was found in the protein complex precipitated with anti-Tax from MT2, MT4 and C8166 cells (Figure 4B, lanes 2–4 compared to 1). This indicated an association of Tax with endogenous LKB1 in these HTLV-1-transformed cells.

Likewise, a protein complex of Tax and SIK1 was also observed in cells expressing Tax and SIK1-WT, but not in cells expressing Tax and SIK-K56M, the kinase-dead mutant (Figure 4C, lanes 2 and 4). Again, Tax seemingly preferred active over inactive SIK1. Additionally, Tax was also found in a protein complex pulled down from cell lysates with GST-SIK2 or GST-SIK3 protein bound to glutathione beads (Figure 4D, lanes 2 and 3 compared to 1). Hence, Tax preferentially associates with active LKB1 and SIKs.

### LKB1 inhibition of Tax is mediated through SIKs, CRTCs and CREB

Although we have shown that LKB1 and SIKs interacted with Tax and inhibited its function, the order of events in the signaling cascade remains to be characterized. Here, we took advantage of various dominant inactive mutants and siRNAs to dissect the LKB1-SIKs-CRTCs-CREB cascade in Tax activation of LTR.

CRTCs and CREB are essential activators of the HTLV-1 LTR and they are regulated by LKB1 and SIKs (Figures 1C and 2D) [7,27]. To formally address whether the suppressive effect of LKB1 was mediated through CRTCs and CREB, we examined whether and how GalCRTC1-M1 and A-CREB might affect the potentiation of Tax activity in LKB1-depleted cells.

**Figure 5:**
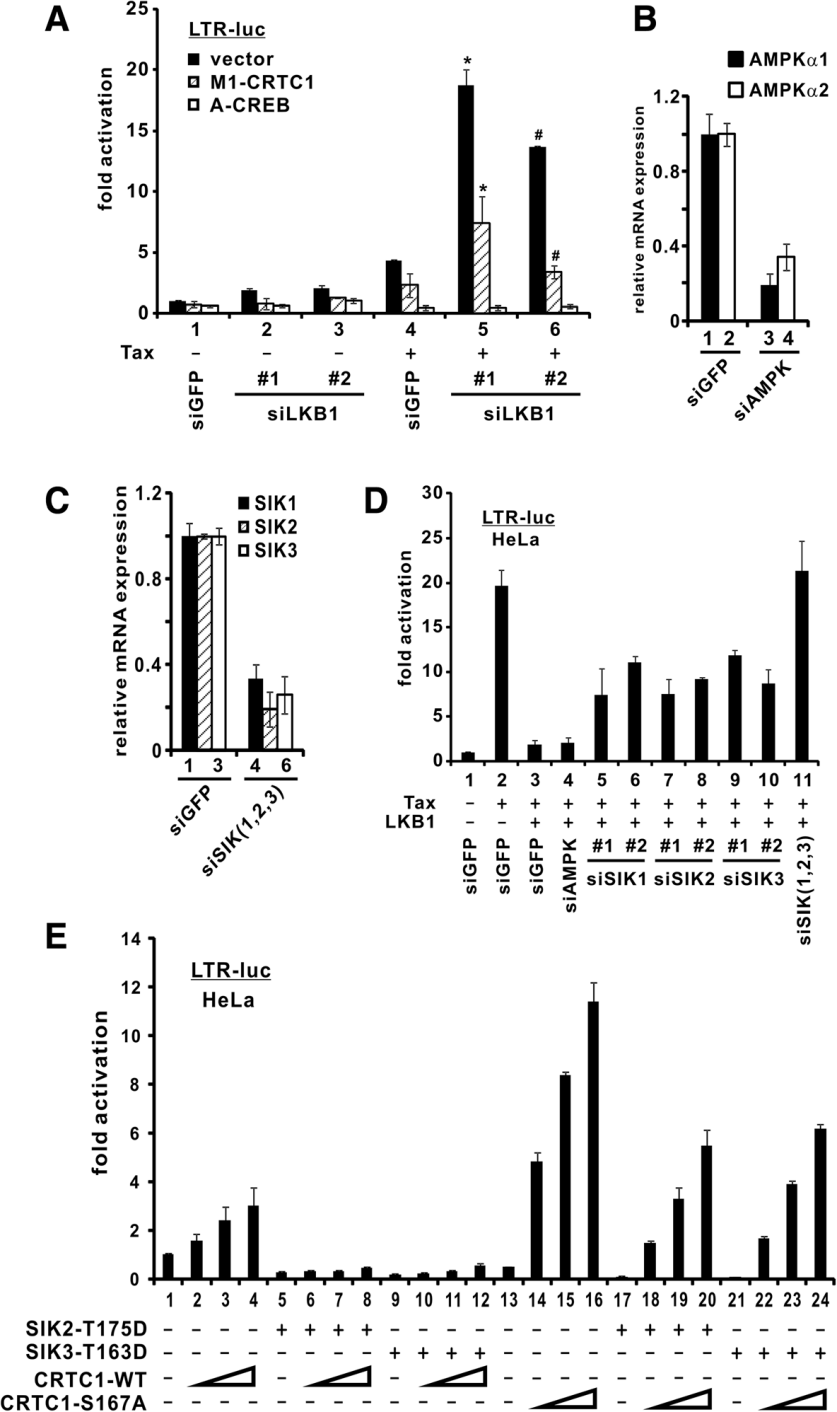
**Requirement of SIKs, CRTCs and CREB in LKB1-mediated suppression of HTLV-1 LTR.** (**A**) Compromising CRTC1 and CREB dampened LTR activity in LKB1-depleted cells. LKB1-depleted HEK293T cells, as shown in Figure 3A, were cotransfected with plasmids pLTR-Luc, pSV-RLuc, pIEX, pM1-CRTC1 and pA-CREB. * and #, the difference between the two groups is statistically significant by two tailed Student’s t test (p = 0.020 and p = 0.0014). (**B**) Verification of AMPK knockdown in HeLa cells. RT-qPCR was performed to analyze AMPK and GAPDH transcripts. The difference between bars 1 and 3 or 2 and 4 is statistically significant (p = 0.00036 or 0.0018). (**C**) Verification of SIK knockdown in HeLa cells. p values were obtained for selected bars (1 and 4: 0.00046; 2 and 5: 0.0046; 3 and 6: 0.00011). (**D**) Endogenous SIKs in HeLa cells are required for LKB1-mediated LTR suppression. AMPKs and SIKs were targeted by independent siRNAs at a concentration of 100 nM. siGFP was used as a negative control. After 36 hrs of knockdown, plasmids pLTR-Luc, pSV-RLuc, pIEX and pCMV-Tag2-LKB1-WT were cotransfected into cells. Cells were harvested 36 hrs after the second transfection. Statistically significant differences exist between group 3 and each of groups 5–11 (p values: 0.041, 0.00042, 0.0057, 0.00028, 0.00021, 0.0018 and 0.0017), whereas the difference between groups 3 and 4 was insignificant (p = 0.735). (**E**) Phosphorylated CRTC1 is required for LTR suppression. HeLa cells were transfected with pLTR-Luc reporter (100 ng), a fixed amount of Tag2-SIK2-T175D (100 ng) or Tag2-SIK3-T163D (100 ng), and escalating amounts of CRTC1 plasmid (18, 37 and 75 ng). Cells were harvested 36 hrs after transfection. The difference between groups 16 and 20 or 16 and 24 is statistically significant (p = 0.00054 or 0.00038).

GalCRTC1-M1 is a truncated mutant of CRTC1 fused to a Gal4 DNA-binding domain and it displayed a potent CRTC1-interfering activity [17]. A-CREB is a dominant inactive form of CREB, which has been widely used [34]. Upon expression of GalCRTC1-M1 or A-CREB, the augmentation of Tax activity ascribed to LKB1 depletion was dampened or abrogated (Figure 5A). Although we cannot exclude other possibilities, these results were generally consistent with the notion that LKB1 requires intact CRTCs and CREB to fulfill its negative regulatory role on Tax.

This result immediately raised a question as to whether SIKs are the intermediate kinases that relay LKB1 signals to CRTCs to regulate LTR activation by Tax. To address this, AMPK and SIK mRNAs were successfully depleted with siRNAs (Figures 5B and 5C). Consistent with our earlier results, LKB1 effectively abolished Tax activation of LTR (Figure 5D, lane 3 compared to 2). Depletion of SIK1, SIK2 or SIK3 individually rescued LKB1-dependent suppression partially (Figure 5D, lane 3 compared to 5–10), whereas knockdown of AMPKα1 and AMPKα2 with one siRNA, which targets a conserved region of both isoforms, did not cause a significant change (Figure 5D, lanes 4 compared to 3). Notably, when we depleted all three SIKs simultaneously, the LKB1-mediated suppression was completely restored (Figure 5D, lane 11 compared to 2 and 3). In keeping with our earlier results (Figure 2E), this further corroborates the notion that SIKs cooperate with each other to mediate the inhibitory effect of LKB1 on Tax activity.

CRTC2 is targeted by SIKs and phosphorylation of CRTCs at conserved serine residues has been suggested as a mechanism of that targeting [27,35]. Taking advantage of an unphosphorylatable CRTC1-S167A, we asked whether the inhibitory activity of SIKs on LTR activation might be mediated through CRTC1 phosphorylation at S167. The experiment was done in the absence of Tax since CRTC1-S167A is a constitutively active mutant that mimics the effect of Tax. Consistent with previous findings [6], CRTC1-WT exhibited modest basal activity on LTR activation (Figure 5E, lane 1 compared to 2–4), whereas LTR activation by CRTC1-S167A was more robust (Figure 5E, lane 13 compared to 14–16). In the presence of dominant active SIK2-T175D or SIK3-T163D, the CRTC1-induced LTR activity was completely blunted (Figure 5E, lanes 5–8 and 9–12 compared to 1–4). In contrast, substantial activation of LTR by CRTC1-S167A was seen in the presence of SIK2-T175D or SIK3-T163D (Figure 5E, lanes 18–20 and 22–24 compared to 14–16), suggesting that SIKs might transmit their inhibitory signal partially through phosphorylation of CRTC1 at S167. On the other hand, mild suppression of CRTC1-S167A activity by SIK2-175D and SIK3-T163D implicated that SIK2 and SIK3 could also regulate CRTC1 through an S167 phosphorylation-independent mechanism. Nevertheless, our collective results suggested that LKB1 operates through SIKs, CRTCs and CREB to effect its suppression on Tax activity.

### LKB1 and SIK1 suppress proviral transcription in HTLV-1-infected cells

**Figure 6:**
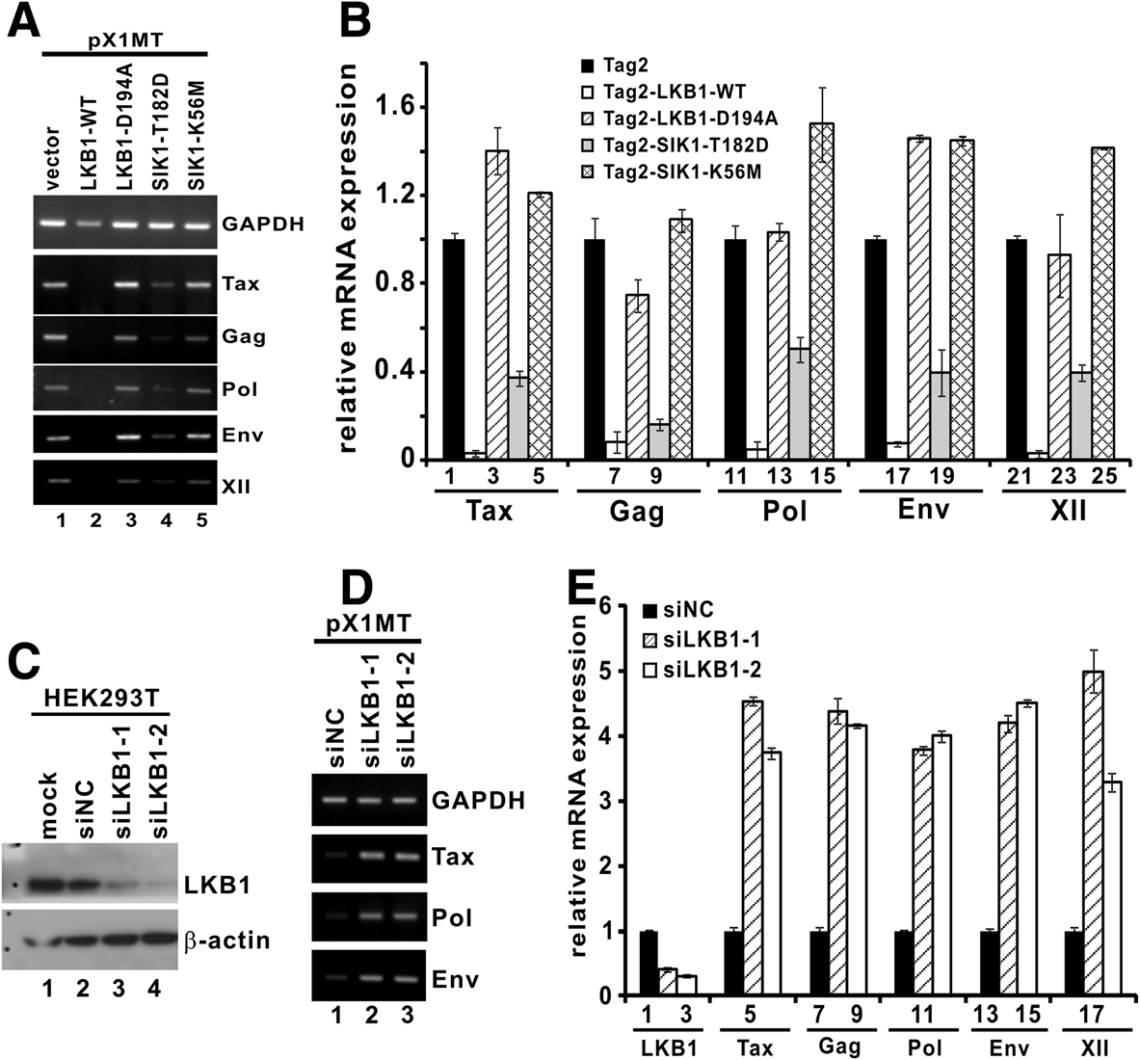
**Modulation of LKB1 and SIK1 influences HTLV-1 proviral transcription.** (**A** and **B**) LKB1 and SIK1 suppressed proviral transcription in a kinase-dependent manner. HeLa cells were co-transfected with pX1MT plus pCMV-Tag2 (vector), LKB1 (WT/D194A) or SIK1 (T182D/K56M). Cells were harvested 72 hrs after transfection. Total RNA was extracted and cDNA was synthesized. Semi-quantitative and quantitative RT-PCR was performed to analyze the relative levels of proviral Tax, Gag, Pol, Env, XII and GAPDH transcripts. The differences between bars 1 and 2, 1 and 4, 6 and 7, 6 and 9, 11 and 12, 11 and 14, 16 and 17, 16 and 19, 21 and 22, as well as 21 and 24 are statistically significant by two tailed Student’s t test (p values: 0.000014, 0.0026, 0.0036, 0.00061, 0.0033, 0.0075, 0.00031, 0.0078, 0.0000023 and 0.00013). (**C**) Verification of LKB1 knockdown in HEK293T cells. Cells were transfected with siRNAs against LKB1 or non-specific control siRNA (siNC) for 72 hrs. Cell lysates were analyzed by Western blotting. (**D** and **E**) Depletion of endogenous LKB1 potentiated HTLV-1 proviral transcription in infected cells. After 36 hrs of LKB1 knockdown, cells were co-transfected with pX1MT for 36 hrs. Cells were harvested and total RNA was extracted for cDNA synthesis. Semi-quantitative and quantitative RT-PCR was performed to analyze the expression of proviral Tax, Pol and Env transcripts. Statistically significant differences exist between bars 1 and 2, 1 and 3, 4 and 5, 4 and 6, 7 and 8, 7 and 9, 10 and 11, 10 and 12, 13 and 14, 13 and 15, 16 and 17, as well as 16 and 18 (p values: 0.000014, 0.00000078, 0.00000021, 0.0000012, 0.0000085, 0.00000014, 0.00000019, 0.00000060, 0.0000025, 0.000000071, 0.000035 and 0.000013).

Above we have characterized the role of LKB1 and SIKs in suppressing Tax activity in LKB1-deficient HeLa cells and LKB1-proficient HEK293T cells. To investigate whether LKB1 and SIK1 might exert a direct suppressive effect on HTLV-1 proviral transcription and replication, we transfected HeLa and HEK293T cells with an HTLV-1 infectious clone termed pX1MT [36]. pX1MT has previously been shown to direct the expression of viral antigens, produce infectious virus, and immortalize primary T cells [36,37]. At 72 hr post-transfection, proviral transcription was monitored by real-time RT-PCR assay. Consistently, the expression of proviral transcripts for Tax, Gag, Pol, Env and XII from pX1MT was significantly repressed in the presence of LKB1-WT and to a lesser extent by the SIK1-T182D dominant active mutant (Figures 6A and 6B), whereas LKB1-D194A or the SIK1-K56M dominant inactive mutant did not affect the expression of proviral transcripts (Figures 6A and 6B). This indicated that the kinase activity of both LKB1 and SIK1 is critical for repression of proviral transcription.

On the other hand, the amounts of proviral transcripts were also examined in LKB1-depleted HEK293T cells. The effectiveness of LKB1 depletion by two independent siRNAs was verified (Figure 6C, lanes 3 and 4 compared to 1 and 2). Consistent with aforementioned results (Figure 3), knockdown of endogenous LKB1 in infected cells augmented proviral transcription (Figures 6D and 6E), indicating a physiological suppressive role of LKB1 on HTLV-1 gene expression during viral infection.

**Figure 7:**
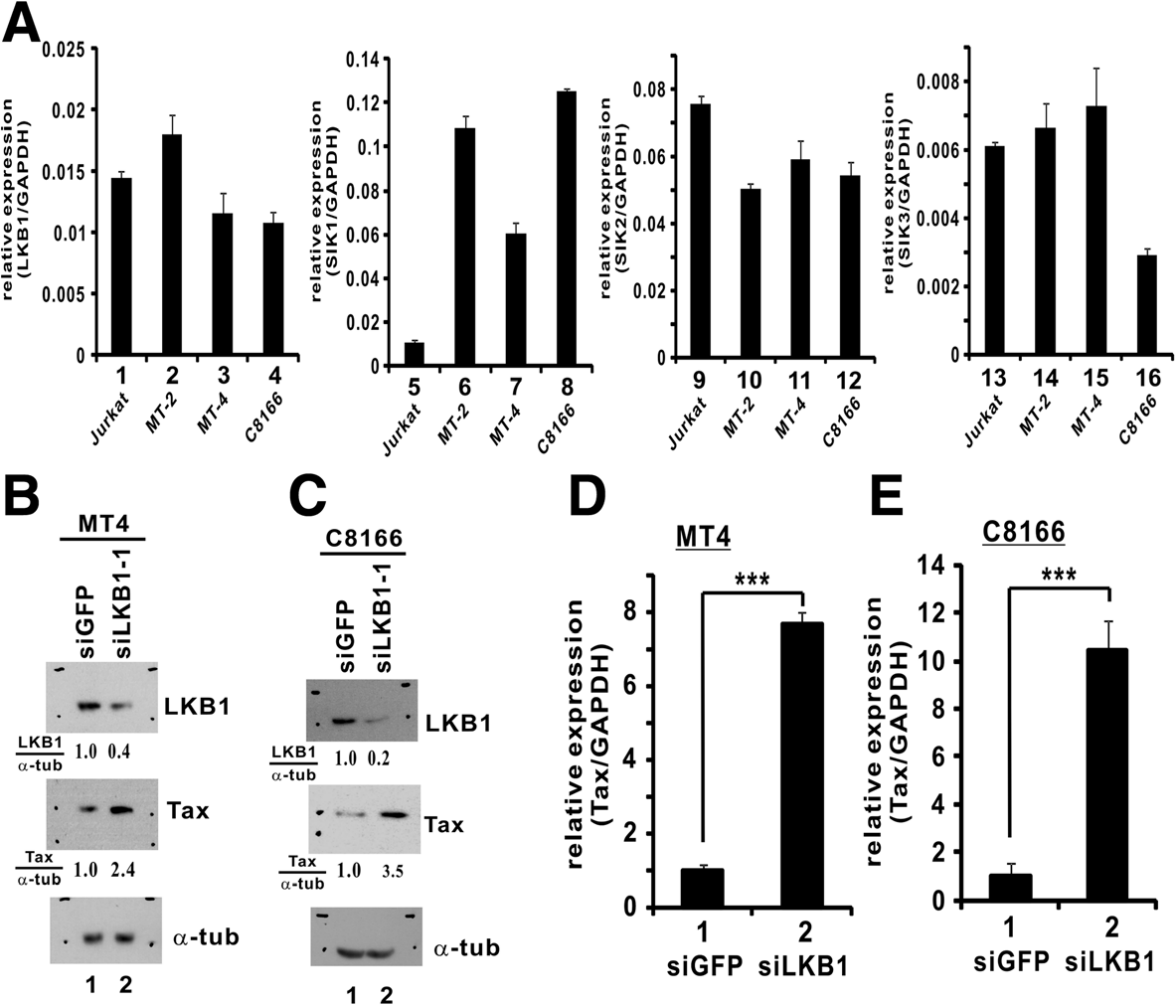
**Knockdown of LKB1 enhances Tax expression in HTLV-1-transformed cells.** (**A**) Expression of LKB1 and SIKs in HTLV-1-transformed cells. RT-qPCR was performed to analyze LKB1, SIK1/2/3 and GAPDH transcripts. Quantitation of mRNA expression was achieved by comparative Ct method^.^ The differences between bars 5 and 6, 5 and 7, 5 and 8, as well as 13 and 16 are statistically significant by two tailed Student’s t test (p values: 0.000011, 0.000082, 0.000000076 and 0.00040). (**B** and **C**) Verification of LKB1 knockdown by siRNA. siLKB-1 was transfected into MT4 and C8166 cells using TransIT-Jurkat transfection reagent (Mirus). Endogenous proteins were analyzed by Western blotting at 48 hrs post-transfection using mouse anti-LKB1, mouse anti-Tax and mouse α-tubulin (α-tub). Relative amounts of LKB1 or Tax normalized to α-tubulin were determined by densitometry and are indicated at the bottom of the panels. (**D** and **E**) Knockdown of endogenous LKB1 enhanced Tax expression in HTLV-1-transformed cells. siLKB1-1 at a concentration of 100 nM was used to deplete endogenous LKB1 in MT4 and C8166 cells. Cells were harvested and total RNA was extracted for cDNA synthesis. Quantitative PCR was performed to analyze Tax and GAPDH transcripts. ***, the difference between the two groups is statistically significant by two tailed Student’s t test (p = 0.00005, MT4; p = 0.001, C8166).

While pX1MT-transfected cells provide a model for acute HTLV-1 infection, HTLV-1-transformed T cells are physiologically more relevant to chronic infection. We found that LKB1 and SIK1/2/3 were expressed in HTLV-1-transformed MT2, MT4 and C8166 cells (Figures 7A). Although it was technically more challenging to transfect siRNAs into MT4 and C8166 cells, we managed to suppress the expression of endogenous LKB1 in these cells with siLKB1-1 using a new transfection reagent (Figures 7B and 7C). We further showed a significant enhancement of Tax expression in LKB1-compromised MT4 and C8166 cells (Figures 7B- 7E). As such, a 2.4- to 10.4-fold elevation of the relative levels of Tax transcript or protein was observed when LKB1 expression was knocked down (Figures 7B- 7E). Collectively, our results supported a physiological role of LKB1 in suppressing proviral transcription in HTLV-1-infected cells.

### Anti-HTLV-1 and antiproliferative effect of metformin

Our observations that LKB1 and SIKs negatively regulate HTLV-1 transcription provide the foundation for rational design and development of molecularly targeted anti-HTLV-1 and anti-ATL drugs. As the first step towards this end, we tested a pharmaceutical activator of LKB1 and SIKs, metformin. As one of the most commonly used antidiabetic drugs, metformin is known to exert its therapeutic effects by activating LKB1 and AMPKs [38,39].

**Figure 8:**
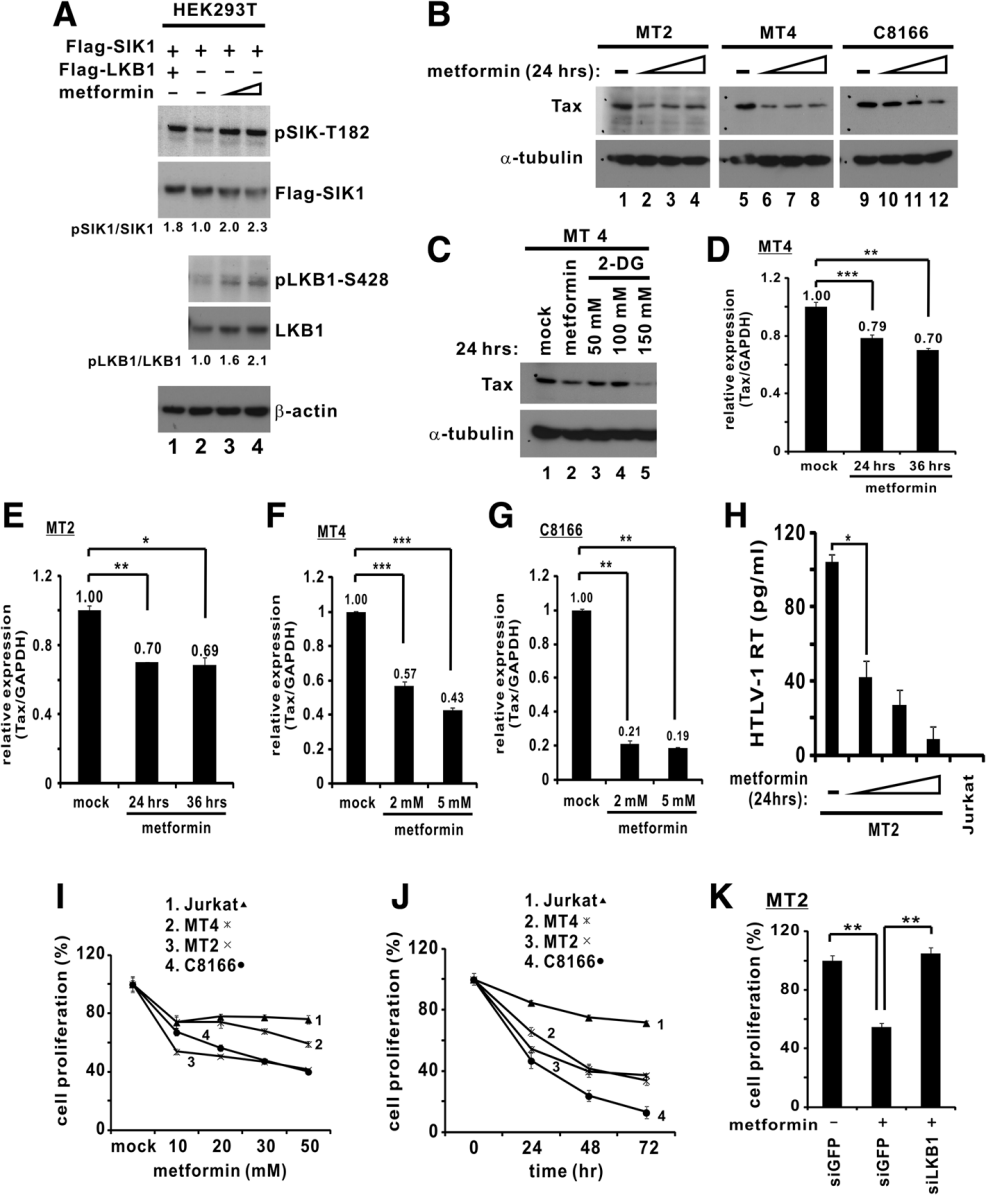
**Metformin activates LKB1-SIK axis and suppresses HTLV-1 proviral gene expression and cell proliferation.** (**A**) Treatment with metformin triggered phosphorylation of LKB1 and SIK1. Confluent HEK293T cells were exposed to metformin (0.5 and 1 mM for 30 min) as described [39]. (**B**) Diminution of Tax protein in HTLV-1-transformed cells treated with metformin. Cells were treated with metformin (5, 10 and 20 mM) for 24 hrs. (**C**) Effect of metformin and 2-DG on Tax expression. MT4 cells were treated with metformin (10 mM) or 2-DG (50, 100, 150 mM) for 24 hrs. (**D** and **E**) Time course of metformin-induced downregulation of Tax mRNA production. Cells were treated with metformin (1 mM) for 24 and 36 hrs. Quantitative PCR was performed to analyze Tax and GAPDH transcripts. Relative Tax mRNA expression in mock-treated cells was taken as 1. *, p = 0.013. **, p = 0.004 (MT4 and MT2). ***, p = 0.00006. (**F** and **G**) Dose dependence of metformin-induced downregulation of Tax mRNA production. Cells were treated with metformin (2 and 5 mM) for 24 hrs. ***, p = 0.002 and p = 0.0003 for MT4; p = 0.0001 and p = 0.00003 for C8166. (**H**) Metformin inhibited HTLV-1 virion production. Cells were treated with metformin (5, 10 or 20 mM). Overnight production of cell-free HTLV-1 virions was assessed by measuring reverse transcriptase activity recovered from live HTLV-1 virus in the culture supernatant with a colorimetric assay kit (Roche). *, p = 0.0059. (**I** and **J**) Metformin inhibited proliferation of HTLV-1-transformed cells. Cell viability was measured by MTT method. (**K**) Metformin-mediated growth inhibition is LKB1-dependent. LKB1-depleted MT2 cells were treated with metformin (20 mM) for 24 hrs. **, p = 0.005, left; p = 0.008, right.

We first verified the activation of LKB1-SIKs axis in cultured cells treated with metformin. Indeed, incubation of HEK293T cells with increasing amounts of metformin caused a progressive increase in the levels of both phospho-LKB1-S428 and phospho-SIK1-T182 (Figure 8A, lane 3 and 4 compared to 2), indicative of the activation of LKB1 and SIKs by metformin. Ectopic expression of LKB1 alone also enhanced SIK1-T182 phosphorylation (Figure 8A, lane 1 compared to 2).

Treatment of three lines of HTLV-1-transformed cells (MT2, MT4 and C8166) with increasing doses of metformin resulted in a progressive reduction in the levels of Tax protein (Figure 8B, lanes 2–4 compared to 1, lanes 6–8 compared to 5, and lanes 10–12 compared to 9). Likewise, treatment of MT4 cells with 2-deoxyglucose (2-DG) also reduced steady-state protein levels of Tax (Figure 8C, lanes 3–5 compared to 1). Previously, 2-DG has been shown to stimulate phosphorylation of LKB1 at both S307 and S428, leading to enhanced activity [39]. To verify that the reduction in Tax expression was attributed to transcriptional repression of LTR in the proviral genome of HTLV-1-transformed cells, we also measured the amounts of Tax transcript. Treatment with three different doses of metformin diminished the levels of Tax mRNA in MT2, MT4 and C8166 cells at two different time points (Figures 8D- 8G). Furthermore, measurement of reverse transcriptase activity associated with live HTLV-1 virus indicated that treatment with metformin suppressed in a dose-dependent manner the production of cell-free HTLV-1 virions released to the culture supernatant of MT2 cells (Figure 8H). These data consistently supported a repressive effect of metformin on HTLV-1 transcription and viral protein expression.

Because HTLV-1 and its Tax protein are thought to drive T cell proliferation and transformation [1,12], we believed it would be of interest to see whether and how inhibition of HTLV-1 transcription and Tax expression by metformin might affect cell proliferation. Indeed, treatment of HTLV-1 transformed MT4, MT2 and C8166 cells with metformin effectively reduced cell proliferation in a dose- and time-dependent manner (Figures 8I and 8J). In contrast, the proliferation of HTLV-1-negative Jurkat cells was not significantly affected. Importantly, depletion of LKB1 in MT2 cells by siLKB1-1 reversed the growth suppressive effect of metformin (Figure 8K), implicating that metformin suppresses proliferation of HTLV-1-positive cells through activation of LKB1.

## Discussion

In this study we provided the first evidence that LKB1 and SIKs negatively regulate HTLV-1 gene expression. We first demonstrated a kinase-dependent suppression of Tax-mediated activation of HTLV-1 LTR by LKB1 and SIKs (Figures 1, 2, 3, 4). We next determined the components of the LKB1-initiated signaling cascade which amplifies and transmits the inhibitory signal to CREB and Tax plausibly through sequential phosphorylation of SIKs and CRTCs (Figure 5). We also documented LKB1-mediated inhibition of proviral gene transcription in HTLV-1-infected cells (Figures 6 and 7). Finally, we demonstrated the anti-HTLV-1 and antiproliferative activity of metformin, a small-molecule agonist of LKB1 and SIKs (Figure 8). Our finding of a previously unrecognized link of LKB1 and SIKs to transcriptional control of HTLV-1 reveals another level of regulation relevant to HTLV-1 pathogenesis and provides new strategies for disease prevention and intervention.

HTLV-1 transcription and replication are critical to the initiation and progression of ATL. Although the exact mechanism of Tax function remains incompletely understood [1], transcriptional activation from the HTLV-1 LTR bears similarities with that driven by cellular CREs. Several lines of evidence in the literature supported the role of LKB1-SIK cascade in the regulation of CRTC activity at cellular CREs. First, LKB1 is a master kinase which activates more than 14 AMPK-related kinases, three of which have been implicated in the regulation of CREB signaling [16]. Second, SIK1 is known to inhibit cAMP-induced transcription [40]. Third, CRTCs interact with CREB and augment CREB activity [41,42]. Finally, SIKs phosphorylate CRTCs and induce their cytoplasmic retention [35,43]. In line with this model, enforced expression of LKB1 in LKB1-deficient HeLa cells led to phosphorylation and activation of SIKs, restoring nucleocytoplasmic shuttling of CRTCs [27]. Some of these findings on cellular CREs are relevant to HTLV-1 LTR.

LKB1 is a highly efficient suppressor of HTLV-1 transcription. Expression of LKB1 in HeLa cells led to an almost complete shut-down of the activity of Tax (Figure 1). In this setting Tax expression driven by a CMV promoter was unaffected by LKB1. Thus, the observed inhibition of LTR activity by LKB1 was not mediated by an indirect effect on Tax. Our findings presented in Figures 2, 3 and 5 are consistent with the notion that LKB1 phosphorylates and activates SIKs, which in turn phosphorylates and inactivates CRTCs, leading to the inhibition of CREB and Tax. Additional loss-of-function and gain-of-function experiments in HTLV-1-infected T cells will provide further support to this model. The S167 phosphorylation-independent mechanism through which SIK2 and SIK3 regulate CRTC1 (Figure 5E) also merits further investigations. Because CREB is required for the transcription of other oncogenic viruses such as hepatitis B virus [44,45], it will be of interest to see whether LKB1 might regulate hepatitis B virus transcription as well.

In addition to LKB1, SIKs were also found to suppress HTLV-1 transcription in this study. To our surprise, AMPKs, which are also activated by LKB1 and can regulate CRTC activity in other systems [28], were not involved in Tax activation of LTR (Figures 2A and 5D). This implies that Tax recruitment of LKB1 substrates has specificity. The inhibition of LTR activation was apparently more prominent when SIK1 + SIK2, SIK1 + SIK3 or SIK1 + SIK2 + SIK3 were expressed in combination (Figure 2E). Consistently, LTR activity was most robust when all three SIKs were compromised (Figure 5D). This functional redundancy and cooperativity of SIKs might be relevant to the regulation of HTLV-1 transcription in different cell types and different stages of viral infection. In this regard, it will be of interest to further investigate the synergistic actions of SIK1, SIK2 and SIK3 in mouse models.

The inactivation of additional tumor suppressors such as p53 might play a role in ATL development and progression [46]. One previous study suggested chromosomal rearrangements at 19p13.3, which harbors the LKB1 tumor suppressor, in some ATL cells [21]. Although LKB1 and all three SIKs were expressed in MT2, MT4 and C8166 cells (Figure 7), it will still be of great interest to see whether genetic and epigenetic inactivation of LKB1 and SIKs might occur in ATL cells and help to further propagate Tax-initiated transformation. Whereas Tax activation of NF-κB is thought to be important in leukemogenesis, CREB signaling is also required for sustained transformation [12]. Leukemogenesis is driven by multiple forces including the targets of CREB, HTLV-1 transcription and Tax [1,12]. In this sense, inactivation of LKB1 and SIKs might promote ATL development through uncontrolled activation of CREB and the HTLV-1 LTR.

Preferential association of Tax with active LKB1 and SIKs (Figure 4) is consistent with the notion that Tax orchestrates HTLV-1 transcription by recruiting both activators and inhibitors. Tax plays a central role in HTLV-1 transcription and it interacts physically with various cellular regulators of the LTR including CRTCs and CREB [7]. The recruitment of active LKB1 and SIKs by Tax plausibly adapts them to CRTCs. This might constitute a negative feedback circuit that controls the magnitude and duration of LTR activation. Plausibly, the expression and activity of LKB1 and SIKs in HTLV-1-infected cells would govern LTR activation in different biological contexts.

The strongest suppression of HTLV-1 LTR by LKB1 and SIKs was observed in transfected HeLa and HEK293T cells (Figures 1, 2, 3). Not to our surprise, the suppressive effects were moderate in T-cell lines, plausibly due to low transfection efficiency (Figures 6, 7, 8). Nevertheless, our gain-of-function and loss-of-function studies performed in pX1MT-transfected and HTLV-1-transformed cells (Figures 6 and 7) consistently supported the notion that LKB1 and SIKs are physiological regulators of HTLV-1 transcription. Hence, pharmaceutical activation of LKB1 and SIKs in HTLV-1-infected cells would serve to counteract HTLV-1 transcription and subsequent transformation. Although HTLV-1 leukemogenesis is a slow process, high proviral loads are a major risk factor for disease progression [3]. Thus, reducing proviral loads with small-molecule agonists of LKB1 and SIKs, such as metformin, might reduce the risk for development of ATL.

Indeed, we demonstrated an anti-HTLV-1 and an LKB1-dependent anti-proliferative activity of metformin in HTLV-1-transformed cells (Figure 8). Further investigations are required to determine the *in vivo* relevance of these findings. Particularly, it will be intriguing to see whether metformin would exhibit anti-HTLV-1 and antiproliferative activity in an animal model. Metformin is one of the most commonly used anti-diabetic drugs. Long-term use of metformin is both well tolerated and highly effective in the activation of LKB1 and downstream kinases [47]. Thus, metformin might be useful not only in patients with ATL, but also in HTLV-1 carriers who are at risk for development of ATL. In this regard, epidemiological studies would be performed to assess retrospectively whether the use of metformin in diabetic HTLV-1 carriers might have reduced the risk for development of HTLV-1-associated diseases.

## Conclusion

Our study defines a negative regulatory role of LKB1 and SIKs in HTLV-1 transcription, which operates through CRTCs and CREB. Our work also provides the proof-of-concept for the utility of metformin, a small-molecule agonist of LKB1 and SIKs, in anti-HTLV-1 and anti-ATL therapy.

## Methods

### Cell culture and transfection

HeLa and HEK293T cells were cultured in Dulbecco modified Eagle medium supplemented with 10% fetal calf serum, 2 mM l-glutamine and 1% penicillin/streptomycin at 37°C in a humidified atmosphere of 5% CO_2_. Jurkat and other HTLV-1-transformed T cells (MT2, MT4 and C8166) were maintained in RPMI1640 medium supplemented with fetal calf serum and penicillin/streptomycin.

HeLa and HEK293T cells were transfected using GeneJuice transfection reagent (Novagen). Jurkat and other HTLV-1-transformed cells were transfected using Lipofectamine 2000 (Invitrogen).

### Plasmids and antibodies

Reporter plasmid pLTR-Luc and expression plasmids for Tax, A-CREB, CRTC1, CRTC1-S167A, CRTC1-M1, SIK2, SIK3, AMPK and AMPK-T172D have been detailed elsewhere [7,17,27,48]. Tax expression plasmid pIEX is driven by a cytomegalovirus (CMV) promoter [49]. The pCAG-Tax-V5 expression plasmid was derived from pIEX. LKB1 cDNA in the pCMV-Tag2-LKB1 expression plasmid was derived from EST clone IRAUp969C0840D. The pCMV-Tag2-SIK1 plasmid was derived from pWZL-Neo-Myr-Flag-SNF1LK provided by Jean Zhao [50]. pCMV-Tag2-SIK2 and pCMV-Tag2-SIK3 were derived from pEBG-SIK2 and pEBG-SIK3, respectively [27].

Mutants for LKB1, AMPKα2 and SIKs were generated by Quikchange Site Directed Mutagenesis kit XL (Agilent). DNA sequencing confirmed that all mutations were successfully introduced. The HTLV-1 infectious clone pX1MT has been described previously [36]. Metformin, 2-deoxy-D-glucose (2-DG), rabbit anti-V5, mouse anti-Flag, mouse anti-β-actin and mouse anti-α-tubulin were obtained from Sigma-Aldrich. Mouse anti-V5 was from Invitrogen. Mouse anti-LKB1 (Ley37D/G6), anti-GST and anti-GFP were from Santa Cruz Biotechnology. Rabbit antibodies against phospho-LKB1-S428 and phospho-acetyl coenzyme A carboxylase-S79 (p-ACC) were from Cell Signaling and Millipore, respectively. Mouse anti-Tax and rabbit anti-phospho-SIK1-T182 have been described [27].

### Reporter assays and protein analysis

The dual luciferase assay and protein analysis were performed as described previously [7,17]. Cells were harvested 36 or 48 hrs after transfection. Transfection efficiencies were normalized to pSV-RLuc (Promega). Three independent experiments were performed and error bars indicate standard deviations (SD). Differences between indicated groups were statistically analyzed by two tailed Student’s *t* test.

### Protein affinity precipitation

HEK293T cells grown in 100-mm petri dish were harvested into 1 ml of immunoprecipitation buffer (20 mM Tris–HCl, pH 7.5, 100 mM NaCl, 0.5 mM EDTA, 0.5% NP-40, 1 mM dithiothreitol, 20 mM β-glycerophosphate, 1 mM sodium vanadate, and 1 mM phenylmethylsulfonyl fluoride). Flag-LKB1/SIK1, V5-Tax or GST-SIK2/SIK3 protein was precipitated from the cleared lysate after a 2-hr incubation at 4°C with mouse anti-Flag (M2, Sigma), mouse anti-V5 (Invitrogen) or glutathione Sepharose 4B (GE Healthcare). Immunoprecipitates were collected with protein G agarose (Invitrogen). Protein complexes were washed three times with immunoprecipitation buffer and subsequently resuspended in sample buffer (50 mM Tris-Cl, 2% sodium dodecyl sulfate, 5% glycerol, 1% β-mercaptoethanol, and 0.002% bromophenol blue). For immunoprecipitation of endogenous Tax, HTLV-1 transformed cells (MT2, MT4 and C8166) were harvested in 1 ml of immunoprecipitation buffer. Cleared lysate was then incubated with mouse anti-Tax.

### RNA interference (RNAi)

HeLa and HEK293T cells were transfected with 100 nM siRNA using Lipofectamine 2000 (Invitrogen). MT2, MT4 and C8166 cells were transfected using TransIT-Jurkat transfection reagent (Mirus). RNAi experiments were performed as described [51]. siRNA sequences are listed in Additional file 2: Table S1.

### Real-time RT-PCR

Real-time RT-PCR was performed as previously described [52,53]. Primer sequences are listed in Addditional file 2 Table S1. Briefly, total RNA was extracted using RNAiso Plus reagent (TaKaRa) and cDNA was synthesized by Transcriptor First Strand cDNA Synthesis Kit (Roche) using random hexamer primers. RNA expression was quantified by real-time PCR using SYBR® Premix Ex Taq™ reagent (TaKaRa) and StepOne™ real-time PCR system (Applied Biosystems). The normalized value in each sample was calculated as the relative quantity of mRNA divided by the relative quantity of GADPH transcript. Quantitation of target mRNA expression was achieved with the comparative Ct method. Relative expression level of target mRNA was calculated from 2^-ΔCt^.

### Cell proliferation assay

Cell proliferation was assayed by the 3-(4,5-dimethylthiazol-2-yl)-2,5-diphenyl-tetrazolium (MTT) method as described [54]. Briefly, 5 × 10^4^ cells treated with 10 μl of MTT solution (5 mg/ml) were measured by a microplate reader (Spectra Max 340, Molecular Devices) at a reference wavelength of 550 nm. Cell viability was calculated as a percentage of the control. In dose–response experiments, cells were treated with 10, 20, 30 and 50 mM metformin for 24 hrs. In time-course analysis, cells were treated with 30 mM metformin for the indicated durations.

### Reverse transcriptase assay

MT-2 cells were seeded at 1 × 10^6^/ml and cultured overnight in the presence of escalating concentrations of metformin (5, 10 or 20 mM). Supernatants were collected and ultracentrifuged for 2 hrs at 200,000 × g. Supernatant from cultured Jurkat cells was used as a negative control. Reverse transcriptase activity was measured using a colorimetric method by the use of a reagent kit from Roche.

### *In vitro* LKB1 activity assay

His-Tax, LKB1 trimeric complex and GST-AMPKα2 (1–312) were bacterially expressed and a kinase assay was performed with purified recombinant proteins as described [48]. Briefly, GST-AMPKα2 (1–312) (5 μg) was incubated with increasing amounts of His-Tax (1, 2 or 4 μg) in AMPK kinase buffer (50 mM HEPES and 0.02% BRIJ35, pH7.0) in the presence or absence of LKB1 trimeric complex (1.5 μg). The reaction was performed in the presence of 20 μM ATP at 30°C for 15 min. Proteins were resolved by SDS-PAGE and detected by Western blotting.

## Competing interests

The authors declare that they have no conflict of interest.

## Authors’ contributions

HMVT and DYJ designed the experiments, analyzed data and wrote the manuscript; HMVT and WWG performed the experiments and analyzed data; CPC and YTS performed pilot study for the project; CMW and KHK analyzed data and provided advice; and YPC and HT provided reagents and advice. All authors read and approved the final manuscript.

## Supplementary Material

Additional file 1: Figure S1 LKB1 kinase activity was unaffected by Tax *in vitro*. GST-AMPKα2 (1–312) (5 μg) was incubated with increasing amounts of His-Tax (1, 2 and 4 μg) in the presence or absence of LKB1 trimeric complex (1.5 μg) in an *in vitro* kinase assay. Proteins were resolved by SDS-PAGE and detected by Western blotting. Click here for file

Additional file 2: Table S1 Nucleotide sequences of RT-PCR primers and siRNAs. Click here for file
