# Supplementary figures and images for: LKB1 tumor suppressor and salt-inducible kinases negatively regulate human T-cell leukemia virus type 1 transcription
Source: Retrovirology. 2013 Apr 11;10:40. doi: 10.1186/1742-4690-10-40 (PMC3640950; doi:10.1186/1742-4690-10-40)

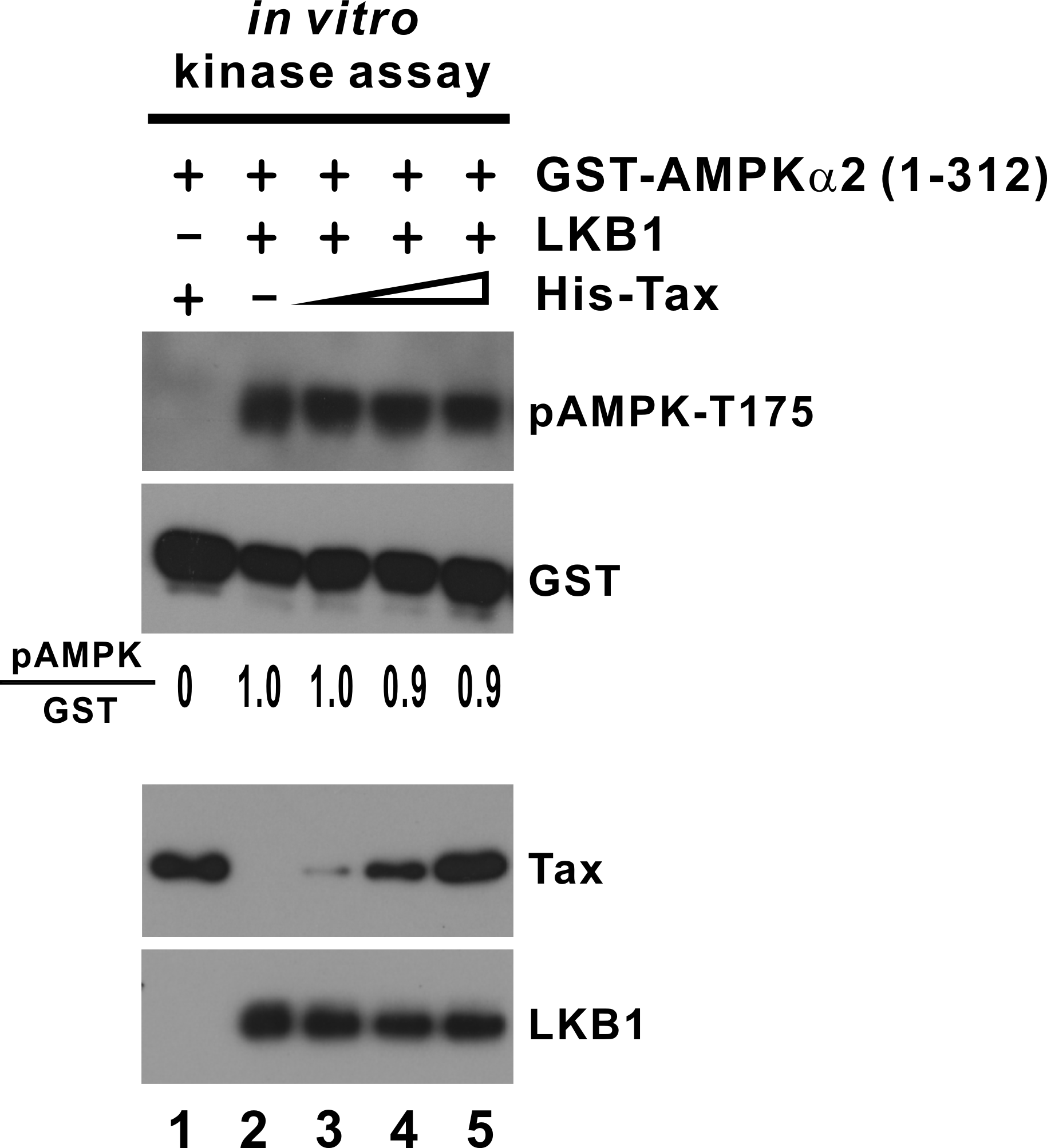

Supplement: Additional file 1: Figure S1 — LKB1 kinase activity was unaffected by Tax in vitro. GST-AMPKα2 (1–312) (5 μg) was incubated with increasing amounts of His-Tax (1, 2 and 4 μg) in the presence or absence of LKB1 trimeric complex (1.5 μg) in an in vitro kinase assay. Proteins were resolved by SDS-PAGE and detected by Western blotting. [file 1742-4690-10-40-S1.tiff]
