# Supplementary material for: LKB1 tumor suppressor and salt-inducible kinases negatively regulate human T-cell leukemia virus type 1 transcription
Source: Retrovirology. 2013 Apr 11;10:40. doi: 10.1186/1742-4690-10-40 (PMC3640950; doi:10.1186/1742-4690-10-40)
Supplement: Additional file 2: Table S1 — Nucleotide sequences of RT-PCR primers and siRNAs. [file 1742-4690-10-40-S2.doc]

**SUPPLEMENTARY MATERIAL**

**Supplementary table 1**

Nucleotide sequences of RT-PCR primers and siRNAs.

| **Primer/siRNA** | **Sequence** |
| --- | --- |
| GAPDH-F | AGAAGGCTGGGGCTCATTTG |
| GAPDH-R | CTGTGGTCATGAGTCCTTC |
| LKB1-F | ACTGAGGAGGTTACGGCACA |
| LKB1-R | CCTGGCACACTGGGAAAC |
| SIK1-F | CAACCTGGGCGACTACGATGAGCA |
| SIK1-R | GGGCGCACTGGGCATTCCGATACT |
| SIK2-F | GCCTTTTCATTTCCAGCATC |
| SIK2-R | GTCTCCAGCCCTTCGTCA |
| SIK3-F | TGGGGAAAATGAGGAATGTG |
| SIK3-R | CGTAGATGGATAGCAAGAGGAG |
| AMPKα1-F | GGAGCCTTGATGTGGTAGGA |
| AMPKα1-R | GACGCCGACTTTCTTTTTCA |
| AMPKα2-F | GCTGGATTTTGAATGGAAGG |
| AMPKα2-R | GAACAGGAACGCTGAGGTGT |
| Tax-F | TCTCACACGGCCTCATACAG |
| Tax-R | ATATTTGGGGCTCATGGTCA |
| Gag-F | CTTTGCTCCTCCCTCGTG |
| Gag-R | TTGCTGGTATTCTCGCCTTA |
| Pol-F | CCTCCTGCCCCGCTTACT |
| Pol-R | GTTGTGGTTGCCCCTTGC |
| Env-F | TGGCGGAGGCTATTATTCAG |
| Env-R | TTGAGGCGTGACACTTCTTG |
| XII-F | CGGATACCCAGTCTACGTGTTTG |
| XII-R | GGGAGTCGAGGGATAAGGAACT |
| siGFP | 5′-GCAAGCUGACCCUGAAGUU-3’ |
| siNC (non-specific control) | 5′-UUCUCCGAACGUGUCACGU-3’ |
| siLKB1-1 | 5′-CCUGCUGAAAGGGAUGCUU-3’ |
| siLKB1-2 | 5′-GCAUUGUGCACAAGGACAU-3’ |
| siSIK1-1 | 5′-CCACACAUCAUAAAGCUUU-3’ |
| siSIK1-2 | 5′-GGACACACACCUGCACAUU-3’ |
| siSIK2-1 | 5′-CCAGAACACCUGUCAGCUU-3’ |
| siSIK2-2 | 5′-CCAGAGACCUGUUCUCUAU-3’ |
| siSIK3-1 | 5′-CCACAGAAUGUGAGCAUUU-3’ |
| siSIK3-2 | 5′-GCUGAAUGCCAACAACUAA-3’ |
| siAMPK | 5′-AUGAUGUCAGAUGGUGAAU-3’ |
